# Tailored Polyelectrolyte Multilayer Systems by Variation of Polyelectrolyte Composition and EDC/NHS Cross-Linking: Controlled Drug Release vs. Drug Reservoir Capabilities and Cellular Response for Improved Osseointegration

**DOI:** 10.3390/polym14204315

**Published:** 2022-10-14

**Authors:** Johanna Ludolph, Holger Rothe, Uwe Schirmer, Katharina Möbus, Christina Behrens, Henning Schliephake, Klaus Liefeith

**Affiliations:** 1 Institute for Bioprocessing and Analytical Measurement Techniques e.V., 37308 Heilbad Heiligenstadt, Germany; 2 Department of Oral and Maxillofacial Surgery, Georg-August-University, 37073 Göttingen, Germany

**Keywords:** polyelectrolyte multilayer, collagen, cross-linking, bone tissue engineering, drug release, drug reservoir, BMP-2, osteogenic differentiation

## Abstract

Polyelectrolyte multilayers (PEM) are versatile tools used to investigate fundamental interactions between material-related parameters and the resulting performance in stem cell differentiation, respectively, in bone tissue engineering. In the present study, we investigate the suitability of PEMs with a varying collagen content for use as drug carriers for the human bone morphogenetic protein 2 (rhBMP-2). We use three different PEM systems consisting either of the positively charged poly-L-lysine or the glycoprotein collagen type I and the negatively charged glycosaminoglycan heparin. For a specific modification of the loading capacity and the release kinetics, the PEMs were stepwise cross-linked before loading with cytokine. We demonstrate the possibility of immobilizing significant amounts of rhBMP-2 in all multilayer systems and to specifically tune its release via cross-linking. Furthermore, we prove that the drug release of rhBMP-2 plays only a minor role in the differentiation of osteoprogenitor cells. We find a significantly higher influence of the immobilized rhBMP-2 within the collagen-rich coatings that obviously represent an excellent mimicry of the native extracellular matrix. The cytokine immobilized in its bioactive form was able to achieve an increase in orders of magnitude both in the early stages of differentiation and in late calcification compared to the unloaded layers.

## 1. Introduction

The layer-by-layer (LbL) coating strategy via polyelectrolytes is a powerful and simple technique to modify and functionalize nearly any kind of surfaces like membranes, discs, hollow tubes, capsules or nanoparticles independently whether they are made of polymers, glasses, ceramics or metals. Moreover, the LbL method has proven to be simple, flexible, effective, inexpensive and reproducible. In other words, the method imposes no restrictions on the size or shape of the substrate and does not require harsh process conditions during the coating process. In this way, an enormous field of applications has emerged over the years, for example in regenerative medicine, as a drug delivery vehicle or as a bioactive coating to render medical implants or prostheses, such as cardiovascular devices, joint prostheses or bone replacement, respectively, making bone augmentation materials more bioactive [1,2,3,4]. Notably, even hydrogels can be modified with polyelectrolyte multilayers (PEM) to enhance cell adhesion, which could be an option especially for synthetic hydrogels with less bioactivity [5]. In addition, the first studies have been published recently in which even native cells were coated with PEM films [6].

From a physico-chemical point of view, polyelectrolytes are polymers that dispose of numerous ionizable groups. In polar solvents, this group starts to dissociate in dependence of the pH, so that these molecules are then electrically charged. Negatively charged polymers are called polyanions and positively charged molecules are called polycations. Special polyelectrolytes that have both positive and negative charges are called polyampholytes or betaines [7].

In the early 1990s, Decher et al. introduced the LbL technique, which is based on the self-assembly of alternating charged polyelectrolytes. Now it is a powerful alternative to self-assembled monolayers or Langmuir–Blodgett deposition. The driving force of the layer formation is the ionic attraction between the opposite charges and the release of counterions [8]. The polyelectrolyte multilayer formation is a complex balance between electrostatic interactions, non-electrostatic interactions and a gain in entropy as the main driving factor [9,10].

It can be ascertained that the original assembly technique for the fabrication of LbL films has not much changed since its seminal introduction by G. Decher. The most extended protocol is the alternated dipping of flat macroscopic substrates into solutions of polyanions and polycations, including the corresponding rinsing cycles between the different dipping steps [11,12,13].

A polyelectrolyte multilayer consists of a predetermined number of double layers, created from alternating the deposition of polyanions and polycations. Such coatings should have certain key features, like biocompatibility, biodegradability and environmental benignity. The physical, chemical and biological properties of PEMs are defined by the used polyelectrolytes and their molecular composition and conformation, the coating architecture, the fabrication process and the possible post-fabrication treatments where required [14].

Exemplarily, in conventional prosthetics, PEMs are used for the modification of implants for bone augmentation or replacement to improve osseointegration or as drug reservoir, e.g., to release antibiotics to prevent implant-associated infections [15,16]. As mentioned above, the application for PEMs is of special importance in the field of TE. In this area, the LbL technique is often used to mimic the natural extracellular matrix for a better tissue integration and biofunctionality. The ECM consists mainly of glycosaminoglycans (GAGs) like chondroitin sulfate, heparin and hyaluronic acid and fibrillary glycoproteins like collagen, fibronectin and laminin. The use of such natural ECM molecules can help to improve the integration of implants by establishing an ECM-analogue microenvironment.

GAGs are linear and polar polysaccharides, which interact more or less intensely with water molecules [17]. Heparin is a highly sulfated GAG in the ECM and is often used to establish drug carrier systems because it has a high binding capacity for cytokines and growth factors. Collagen is the most common ECM component. It has a defined 3D structure with numerous cell-binding sites. Poly-L-lysin (PLL) is a positively charged polyelectrolyte and often used in PEM films to improve the cell adhesion capability and biocompatibility of surfaces due to the Coulomb interaction. In this study, we have investigated PEMs composed of PLL, heparin and collagen in view of their effect on MC3T3 cell differentiation. MC3T3 pre-osteoblasts represent an established model for in vitro osteoblast differentiation and ECM signaling.

In the last decade, the influence of substrate stiffness on the behavior of biological systems is increasingly becoming the focus of many research activities. Therefore, PEMs are often cross-linked after their assembly to modulate the mechanical properties. A detailed overview of different cross-linking strategies is given in the excellent review of Ghiorghita (2019) [18]. The most common method is cross-linking via EDC (1-ethyl-3-[3-dimethylaminopropyl] carbodiimide hydrochloride) and NHS for PEMs with appropriate functional groups, such as amine and carboxyl groups in aqueous solutions. Cross-linkers like EDC are used to couple carboxyl and amine groups in order to get amide bonds [19]. Ren and coworkers showed that an increase in the EDC concentration is directly proportional to the mechanical stiffness of a (PLL/PA)_12_ PEM. They generated PEMs that showed Young’s moduli from 3 kPa for native films, 100 kPa for low cross-linked films to 400 kPa for highly cross-linked films. Obviously, a higher cross-linker concentration also enhances the myoblast adhesion on the stiffest PEMs and well-defined focal adhesions were observed [20]. Schneider et al. (2007) cross-linked a PLL/HA and a CHI/HA PEM with EDC/NHS. The cross-linked film was about 10 times stiffer than the native non-cross-linked counterpart (native PLL/HA 20 kPa, cross-linked PLL/HA 250 kPa; native CHI/HA 15 kPa, cross-linked CHI/HA 159 kPa). For EDC and NHS concentrations of 70 mg/mL, respectively, 22 mg/mL were employed in a 0.15 M NaCl solution at a pH of 4.5 in order to deposit a thin film for 18 h at 4 °C [21].

For cross-linking PEM films made of collagen, it should be mentioned that the EDC/NHS chemistry can change the structure of the collagen while cross-linking. This may lead to a change in the cell adhesion behavior. The group of Cameron et al. observed a decreasing cell adhesion with an increasing EDC/NHS concentration for cross-linking [22,23]. The reason is that EDC inhibits the cation-dependent cell adhesion to collagen, because the binding sites for the collagen binding integrins α_1_β_1_, α_2_β_1_, α_10_β_1_ and α_11_β_1_ are changed [22].

A possible alternative to cross-link PEMs is given by the use of glutaraldehyde (GA). The amine groups react with the GA and form an imide bond, resulting in a cross-linked PEM. This method was successful used in the field of environmental technology to cross-link PEMs on membranes for water treatment facilities or to improve the stability of PEM microcapsules for pharmacological purposes [19,24,25]. Another option of an increasing importance seems to be the use of genipin to cross-link PEMs. Genipin is a natural cross-linking agent which is found in plants, and it has been quite efficient in protein cross-linking. It can be an interesting alternative for cross-linking with EDC or glutaraldehyde, which often changes the physicochemical properties of the PEM film while cross-linking. In tissue engineering, genipin has shown beneficial effects like the control of inflammation or the enhancement of fibroblastic cell attachment [26]. Chaubaroux et al. (2012) assembled a film of collagen and alginate cross-linked with genipin to stabilize the film at physiological pH values. Compared to films cross-linked with glutaraldehyde, the genipin cross-linked film shows the same structure as a non-cross-linked film. They also observed a satisfying degree of proliferation of HUVECs. The cells grow more confluently and a better spreading behavior was observed on genipin cross-linked films in comparison with a glutaraldehyde cross-linked film [26].

It is well known that growth factors like hBMP-2 can be sequestered within the ECM by means of binding to the protein moieties or to charged glycosaminoglycans, for example, to establish growth factor gradients or to build up a growth factor reservoir for later use. Among the many advantages of LbL assembly are the mild aqueous fabrication conditions and the associated possibility of incorporating cytokines or growth factors without the risk of destabilizing these compounds. This circumstance, along with the potential multivalent charge interactions between the ionized groups of the polyelectrolytes and the charged drugs, renders PEMs as a versatile technology platform to immobilize bioactive agents.

These components can be loaded as direct building blocks during the assembly process or by post-diffusion into the final multilayer system. The subsequent release is controlled by the molecular structure and the charge of the different polyelectrolytes [17], leading to a more or less defined permeability of the polyelectrolyte multilayer in dependence of an external stimuli such as the pH, ionic strength, temperature, etc. [18]. The study of Dash et al. (2010) gives an overview of the mathematical models used to describe the release kinetics of drug delivery systems [27]. The most commonly used kinetic models for PEM films are based on Fick’s law of diffusion and the well-known algorithms of Korsmeyer–Peppas and Higuchi [28].

In bone tissue engineering, BMPs play an important role. The molecular weight of hBMP-2 is 32 kDa and the isoelectric point is given with a pH value of 8.5. In solutions with lower pH values, it is slightly positively charged and can therefore also be electrostatically incorporated into PEMs. BMP-2 is a cytokine and a growth factor and, as such, it is involved in bone healing and different phases of bone repair as a kind of osseous inductor [29].

It should be noted that the clinical dose for therapeutical purposes is relatively high, ranging from 0.1 to 0.5 mg BMP-2/kg body weight [30]. BMPs have a conserved binding site with a specific affinity for sulfated glycosaminoglycans, which renders GAGs as a promising BMP-2 delivery tool. Especially, heparin has a high dissociation constant *K_d_* of 20 nM for BMP-2 and can also enhance the BMP-2 activity [30]. An N-terminal sequence of approximately 13 amino acid residues mediates the binding of the cytokine to heparin [31].

Numerous studies were published which show the positive effect of hBMP-2 in the process of bone formation after drug release. MacDonald et al. (2011) have fabricated a special PEM made of Poly2, a synthetic, intrinsically tunable cationic polymer, and chondroitin sulfate to be able to functionalize 3D scaffolds based on a polycaprolactone/β-tricalcium phosphate (PCL/BTCP) co-polymer blend. The BMP-2 was introduced directly in the coating process. Subsequently, the following tetralayers were obtained: [Poly2/chondroitin sulfate/BMP-2/chondroitin sulfate]_n_, where n is the repeating unit of the tetralayer (*n* = 100). The films show distinct release profiles. A linear release is observed for the first 2 days, where 80% of the BMP-2 is released. In the additional 2 weeks, the last 20% are slowly released. In total, the scaffolds released around 11 mg of BMP-2. The bioactivity of the coating was tested in vitro with MC3T3 E1 subclone 4 pre-osteoblasts and in vivo with sixteen 350–400 g male Sprague Dawley rats. The experiments show a significantly better bone formation for coatings that contain BMP-2 [32]. Guillot and coworkers (2016) investigated the osseointegration of titanium implants (Ti-6Al-4V) and poly(etheretherketone) (PEEK) implants coated with a PEM film which was loaded with BMP-2. The PEM consist of 24 double layers of poly-L-lysine and hyaluronic acid. To enhance the PEM film adhesion on the implant, a Polyethyleneimine layer was introduced at first. After the multilayer generation, the films were cross-linked with EDC/NHS. The coated implants were incubated with 100 µg/mL BMP-2 for 90 min at 37 °C. Uncoated and coated (total dose of 9.3 µg BMP-2) screw implants were inserted in the femoral condyles of rabbits and the osseointegration was compared after 4 and 8 weeks. Surprisingly, the bone-to-implant contact and the bone area around the implant were significantly lower for the coated implants than for the uncoated implants. This impressive study shows that it is important to choose a proper dose of the growth factor, otherwise localized and temporary bone impairment can appear [33].

As already mentioned, both the absolute amount and the release time/kinetic represents an important aspect of drug release. Thus, numerous works have been performed to try to develop biomimetic delivery systems that control the localization, the release time and the kinetics as well as the spatial and temporal release patterns [34]. The goal is to achieve a long-term delivery system that releases the drug component precisely when the cells remodulate the PEM system.

In this context, an intensive scientific debate is taking place about the preference of physiological drug reservoir systems vs. a drug release system. Most release systems start with an initial burst release where almost 80% of the loaded drug is released in the first few hours. However, the adhesion and healing process in vivo takes a significantly longer time. Furthermore, the burst release of cytokines can lead to a very high concentration in the tissue and subsequent tissue inflammation and ectopic ossification [30,33]. Therefore, a drug reservoir, which releases the cytokine just when the cells remodulate the artificial matrix, seems to be extremely attractive.

In this study, we investigated three different polyelectrolyte multilayer systems consisting of PLL, heparin and collagen. The amount of collagen in the layer systems was variable. Each layer system was gradually cross-linked by EDC/NHS chemistry to generate polyelectrolyte multilayers with a different stiffness and defined release kinetics. The PEMs were loaded with hBMP-2 and the total amount in the coating as well as the release as a function of time and in dependence of PEM composition and a degree of cross-linking was investigated. The experiments aim to comparatively quantify the balance between hBMP-2 release and hBMP-2 sequestering within the PEM in terms of bioavailability and the effect on osteoprogenitor cell differentiation.

## 2. Materials and Methods

### 2.1. Coating and Cross-Linking

**Figure 1:**
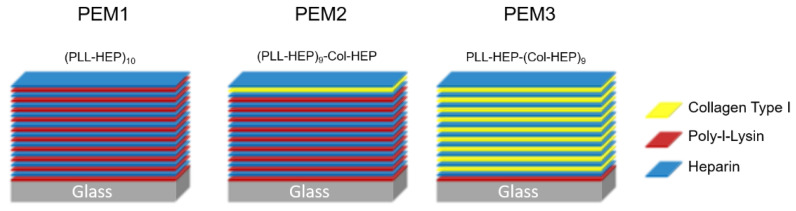
Architecture of the investigated LbL coatings.

Chemicals and reagents were used without further purification, unless stated otherwise. PEM films were assembled from poly-L-lysine (Sigma-Aldrich GmbH, Taufkirchen, Germany, P2636, PLL, 30–70 kDa), heparin (Sigma-Aldrich GmbH, Taufkirchen, Germany, H3393, Hep, from porcine intestinal mucosa) and collagen (ibidi GmbH, Gräfelfing, Germany, 50202, rat tail collagen type I). The polyelectrolytes for PLL–Hep films were dissolved in a Na-acetate buffer (20 mM, pH 4.5) at a concentration of 1 mg/mL. For the deposition of Col-Hep multilayers, the polyelectrolytes were dissolved in 5 mM Acetate (pH 3.5) at a concentration of 1 mg/mL. The film construction was performed automatically by employing a dipping robot (DR3, Riegler & Kirstein GmbH, Potsdam, Germany). Briefly, the cleaned substrates were first dipped into the polycation solution (PLL) and left to adsorb for 5 min. After that, the samples were washed three times in double-distilled water to rinse the surface from the unbound polyelectrolyte. Subsequently, (Hep) was deposited in the same manner. For the (Col-Hep) film construction, the substrate was dipped for 30 min into the solved polyelectrolyte. The rinsing steps here were the same as described for the (PLL-Hep) films. Each cycle was repeated until the desired number was reached of double layers and film architecture. All samples were rinsed in double-distilled water and air dried in a gentle stream of pressurized air. Figure 1 illustrates the coating architecture of the three investigated multilayer systems.

The cross-linking was done using EDC/NHS chemistry at five different EDC concentration levels (0, 5, 25, 100 and 200 mg/mL) in order to obtain peptide bonds within the polyelectrolyte multilayers between the carboxylic groups of the poly-anion (heparin) and the amine groups of the poly-cations (poly-L-lysin; collagen). The EDC stock solution (400 mg/mL) was prepared in ice cold 0.15 M of NaCl with a pH value of 5, and from this the solution was further diluted in separate volumes until the two-fold target concentration was reached. Finally, these two-fold pre-dilutions were further mixed in an equal volume with a 22 mg/mL NHS solution. Each cross-linking reaction was done in 1 mL EDC/NHS-solution in 24-well plates. All working steps were performed on ice with pre-cooled buffer and all samples were incubated over night at 4 °C. After the cross-linking, all samples were rinsed three times with 0.15 M NaCl, pH 8 at room temperature. Between each washing step, the samples were incubated for 1 h. Finally, the samples were rinsed with water and allowed to dry in air at room temperature. For sterilization the samples were placed for 30 min under UV light.

### 2.2. Loading of BMP-2

For the loading of the cytokine in the PEM films, the BMP-2 (PeproTech Germany, Hamburg, Germany) was diluted to a final concentration of 75 µg/mL. The loading was performed in a 24-well plate, where in each well a coated glass disc was placed. An aliquot of 200 µL of the BMP-2 solution was added to each sample per well and incubated over night at 4 °C. The cytokine solution was replaced and the samples were rinsed three times with double-distilled water for 5 min. The samples were then air-dried and stored at 4 °C until further use.

### 2.3. Release Kinetic

All coated glass discs were placed into a 24-well plate and incubated with 400 µL of DMEM with 1% Penicillin/Streptomycin at 37 °C and 5% CO_2_. The medium was collected and replaced by a fresh medium after 1 h, 2 h, 4 h, 6 h, 8 h, 10 h, 24 h, 48 h, 96 h and 192 h.

There are some advanced approaches that can be used to model the loading quantities in polyelectrolyte multilayers, e.g., based on ellipsometry data [35]. In the present study, however, both the release and the loading quantities were high enough to realize the quantification directly via a commercial fluorescence kit. The initial loading capacity as well as the release of rhBMP-2 were measured using a Human/Murine/Rat BMP-2 Standard TMB ELISA Development Kit (PeproTech Germany, Hamburg, Germany), according to the manufacturer’s instructions. The loading amount was calculated by a measurement of the growth factor concentration of the loading solution before and after the incubation. All measurements were done in triplicate on three samples, respectively.

### 2.4. Cell Culture Tests

Mouse fibroblasts MC3T3 cells were obtained from Leibniz Institute DSMZ (Leibniz Institute DSMZ German Collection of Microorganisms and Cell Cultures GmbH, Braunschweig, Germany) and cultured in a proliferation medium, which consisted of α-MEM with 10% FBS and 1% Penicillin/Streptomycin (all the medium components were obtained from PAN-Biotech, Aidenbach, Germany). The cells were subcultured prior to reaching a 70–80% confluence. For the differentiation into osteoblasts, the culture medium was changed to a differentiation medium, which consisted of α-MEM, 10% FBS, 1% Penicillin/Streptomycin and additionally 10 mM of β-Glycerolphosphate and 0.05 mM of ascorbic acid (Sigma Aldrich GmbH, Taufkirchen, Germany).

### 2.5. Proliferation Assay—XTT

For the analysis of proliferation and the viability of the MC3T3 cells on different coatings, an XTT assay was employed. This is a colorimetric assay using XTT (2,3-Bis-(2-Methoxy-4-Nitro-5-Sulfophenyl)-2*H*-Tetrazolium-5-Carboxanilide), a yellow tetrazolium salt, which is reduced in the presence of an electron coupling reagent (5-Methylphenazinium-methasulfat, PMS) to an orange formazan dye by metabolically active cells. The formazan salt is soluble in aqueous solutions and can be directly quantified using a spectrophotometer. For the assay, the MC3T3 cells were cultured with a proliferation medium on the coated discs in 24-well plates. At the sample points, the culture medium was replaced by a 300 µL fresh proliferation medium, then 150 µL of XTT solution (1 mg/mL XTT in RPMI medium and XX mg/mL Electron coupling reagent) were added and incubated at 37 °C for 4 h. After the incubation period, the absorbance of the supernatant was measured using an ELISA plate spectrometer at a wavelength of 405 nm. The cells were washed twice in PBS and then further cultivated in a proliferation medium.

### 2.6. ALP Assay

The cells were seeded at a density of 10,000 cells/cm^2^ on the samples in 24-well plates. The alkaline phosphatase (ALP) activity is a marker for early osteogenic differentiation. To measure the ALP activity, the cells were washed twice with PBS and then lysed with a lysis buffer (50 mM Tris-HCl pH 8.0, 150 mM NaCl, 1% Triton X-100, 100 µg/mL PMSF) for 30 min on ice. One capsule of phosphatase substrate (Sigma Aldrich GmbH, Taufkirchen, Germany) was dissolved in 25 mL of ddH_2_O and then mixed in equal parts with an alkaline buffer (Sigma Aldrich GmbH, Taufkirchen, Germany).

An aliquot of 10 µL of each sample was added to a 96-well plate on ice. The plate was placed to a Thermoblock at 37 °C and 100 µL of the phosphatase solution was added. After 15 min of incubation, the reaction was stopped with 100 µL of 0.5 M NaOH. The absorbance at 405 nm was analyzed with an ELISA reader. For the standard curve, a Nitrophenol solution (Sigma Aldrich GmbH, Taufkirchen, Germany) was diluted with 0.02 M NaOH and used to calculate the units of the enzyme activity.

### 2.7. Matrix Calcification—Alizarin Red S Assay

The calcium deposition and matrix calcification are later markers of osteogenic differentiation and can be analyzed with an Alizarin Red Assay. The cells were seeded at a density of 10,000 cells/cm^2^ on samples in 24-well plates and incubated for 28 days at 37 °C. Then they were washed twice with PBS and fixed with 4% PFA for 60 min. A staining solution was prepared with 2% Alizarin Red S in ddH_2_O at a pH of 4.2. A volume of 200 µL of staining solution was added to each sample and incubated for 45 min in the dark. The samples were washed four times with ddH_2_O for 5 min. For destaining, the samples were incubated with 10% acetic acid for 30 min. The absorbance was analyzed at 405 nm with an ELISA reader.

### 2.8. Microscopy

The cells on the samples were rinsed twice with PBS and then were fixed with 4% PFA for 20 min. The cells were stained with Phalloidin590 (MoBiTec GmbH, Göttingen, Germany) and Hoechst33258 (Thermo Fisher Scientific GmbH, Bremen, Germany) according to the manufacturer’s instruction and analyzed with a Confocal Laser Scanning Microscope (Carl Zeiss Microscopy GmbH, Oberkochen, Germany).

### 2.9. Statistical Analysis

All the results are shown as mean values ± standard deviation. The results of a one-way ANOVA test can be found in the Appendix A

## 3. Results and Discussion

We examined three different layer systems based on the three different polymers: heparin (Hep), poly-L-lysin (PLL) and collagen (Col). The polyelectrolyte multilayers were built up using the LbL technique until a final film architecture consisting of ten double layers was achieved. PEM1 consists of ten double layers (PLL-Hep)_10_. PEM2 has nearly the same structure as PEM1, however, in the last double layer, PLL was replaced by collagen (PLL-Hep)_9_-(Col-Hep)_1_. For the PEM3 system, this replacement strategy was continued for all double layers except for the first double layer, where a strong polycation is needed to guarantee a stable adhesion on the substrate. Thus, the final PEM3 structure is given with the following coating sequence (PLL-Hep)_1_-(Col-Hep)_9_.

For further information, these three layer systems were thoroughly investigated by the means of a specifically adapted spectrum of the physicochemical methods in order to obtain precise data on the multilayer architecture and morphology. A detailed presentation and discussion of the results with special attention to the most important factors of influence such as the stiffness, layer thickness, zetapotential, roughness parameter and the intrinsic and extrinsic charge compensation, respectively, has already been published by the authors [33]. Table 1 summarizes the relevant data on the respective physicochemical investigations.

As can be seen, the layer composition and the post-cross-linking hardly shows a clear influence on the wettability of the layer systems. The water contact angle is below 30° in the very hydrophilic range. Only highly cross-linked coatings (200 mg/mL EDC/NHS with PEM-1; 100 mg/mL and 200 mg/mL EDC/NHS with PEM-3) particularly show a slight increase in the water contact angle to 40° to 50°. However, clear trends cannot be derived from the wettability, which is why a significant influence of the wetting on the release or the binding behavior of the cytokine can be excluded.

The same applies to the roughness determined by AFM. Since the specific surface of the layer systems is relevant for a release of the cytokine, the Sdr value was determined in addition to the Ra value. Slight trends are recognizable from the results. With increasing the cross-linking, the roughness of the PEM-1 and PEM-2 systems increases slightly. This trend is not observed for the PEM-3 system. However, the changes in the specific surface area for all layer systems are below 1%, so an influence of the roughness on the release behavior is very unlikely.

The FT-IR measurements show an increase in the amide-I bands, which demonstrates the successful cross-linking of the coatings using EDC/NHS. For the PEM-2 and PEM-3 coating systems, an expected stiffening due to cross-linking could also be demonstrated. The PEM-I system produces a layer thickness that is too low to reliably determine data on the stiffness using AFM, as an influence of the substrate material cannot be ruled out.

Nevertheless, due to the cross-linking and the resulting compacting of the layer systems, both a reduction in the loading capacity and a delay in the release can be expected.

### 3.1. Loading Capacity and Release Kinetics

The different PEM systems were loaded with rhBMP-2 and the release of the cytokine was observed over 192 h. The amount of released rhBMP-2 was measured by an ELISA and the results were accumulated for each time point.

**Figure 2:**
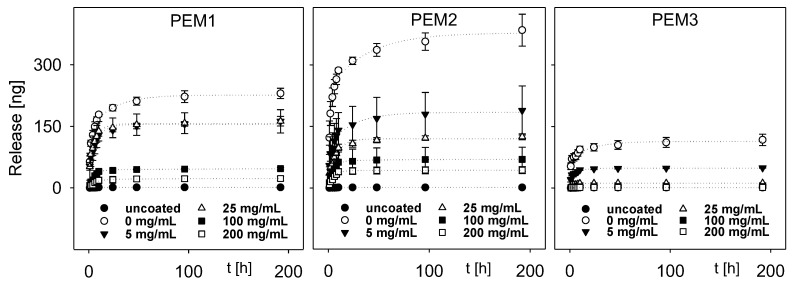
Release kinetics of the three coatings in dependence of the degree of cross-linking, dotted lines are to guide the eyes.

Figure 2 shows the resulting release profiles for each PEM system. Especially for the coatings PEM1 and PEM2, a release of the cytokine can be observed mainly within the first 48 h. The collagen-rich system PEM3 shows a burst release within the first 10 h.

As expected, the multilayer films PEM1 and PEM2 show very similar release characteristics due to the comparable layer architecture. Both multilayers consist of a backbone of nine PLL–HEP double layers and in contrast to PEM1, PLL was substituted by collagen within the top layer of PEM2. A detailed consideration shows a slightly increased release of rhBMP-2 from the PEM2 system in comparison to the PEM1 system. Probably, this effect could be explained by the higher layer thickness of the coating. As we have shown elsewhere, the substitution of PLL by collagen results in a sudden increase in the coating thickness, which is a result of an incomplete charge compensation by the weak poly-cation collagen [36].

Apparently, this change in the layer architecture may lead to a slightly looser, open-porous layer architecture and a decrease in layer stiffness, which enables a higher release.

**Figure 3:**
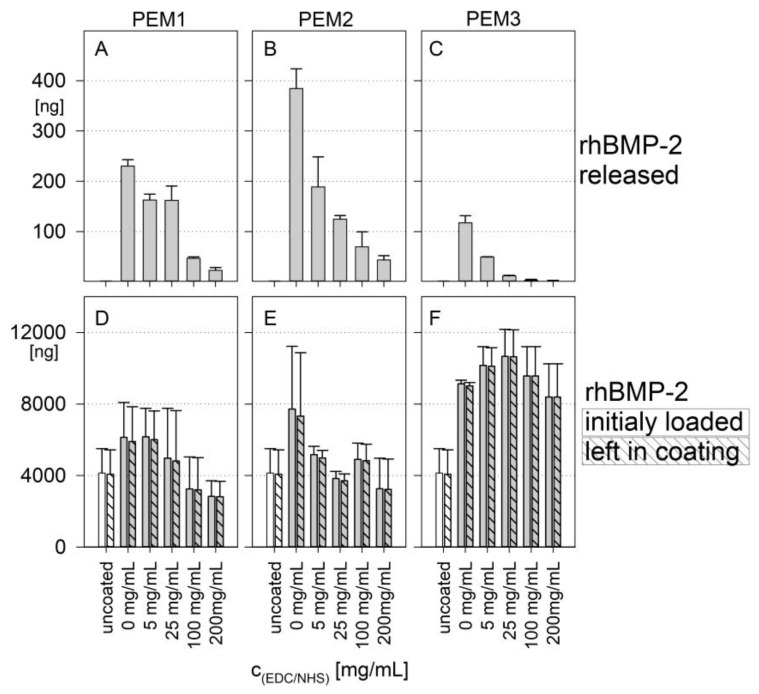
(**A**–**C**): Amount of the released rhBMP-2 after 192 h; (**D**–**F**): amount of rhBMP-2 initially loaded (clear bars) and left in coatings (hatched bars) in dependence of the degree of cross-linking. Results of a one-way ANOVA test can be found in the Appendix A in Figure A1 and Figure A2.

A completely different release behavior was observed for the PEM3 multilayer. The release of rhBMP-2 was significantly decreased. In addition to the simple quantitative effect caused by the thickness difference, it can be assumed that, caused by the weak cationic character of collagen, heparin is much more freely available within the PEM3 multilayer, which can act as a kind of drug reservoir for rhBMP-2. Consequently, this (i) increases the total loading capacity of the polyelectrolyte multilayer and (ii) inhibits the release due to the high binding capability of heparin. For a better comparability, the total release and the absolute amount of the initial rhBMP-2 loading are shown in Figure 3. It is remarkable that the total release is more than one order of magnitude lower than the initial loading of the coatings with cytokine. In percentage terms, the release amounts for PEM-1 range from ~4% for the not cross-linked coatings down to ~1% for the highest degree of cross-linking. The PEM-2 system shows a respective percentage release between ~5% down to ~1.5%, while the PEM-3 system shows a significant lower percentage release of ~1.3% down to almost zero.

Furthermore, it becomes clear that the loading capacity of the coatings (Figure 3D–F) is only slightly influenced by the cross-linking. Instead, it can be inferred that the loading capacity of the coatings is primarily determined by the coating architecture. With that in mind, it is important to mention that the PEM3 multilayer, which is composed of heparin and collagen, shows a loading capacity of around 8000 ng rhBMP-2, whereas the multilayers PEM1 and PEM2, which have a backbone consisting predominantly of PLL–HEP, show a significantly reduced loading capacity of around 4000 ng.

Obviously, drug delivery can also be targeted by a defined variation of the degree of cross-linking. A higher cross-linking of the multilayer films initially leads to a higher stiffness of the coatings and causes a significant inhibition of the release of BMP-2 [36]. This observation is particularly true for the PEM3 multilayer, as could be shown by the near zero order release kinetic for higher degrees of cross-linking (Figure 3C).

A similar phenomenon was observed by Crouzier et al. in 2009 who investigated PEM systems containing poly-L-lysine as cationic and hyaluronic acid as anionic polyelectrolytes. The authors incorporated BMP-2 in the layer and determined also a low initial release of the cytokine. The BMP-2 remains predominately in the layer and is still available for the cells in its bioactive form [37].

Nevertheless, numerous studies were focused on GAG-based PEMs as drug delivery system for cytokines and growth factors, especially with heparin and BMP-2. These investigations were usually carried out with the aim to establish a controlled and localized or site-specific drug delivery over a long period of time [17,38]. Introducing ECM-analogue polyelectrolytes like hydrogels or heparin leads to an additional protective function for cytokines, especially BMP-2, and can slow down the release rate. In hyaluronic acid hydrogel particles which are decorated with heparin, the loading capacity and the release of BMP-2 is strongly influenced by the heparin. The heparin changed the initial burst phase of the cytokine release to a near zero order release kinetic [39]. Ao et al. (2020) observed that the incorporation of heparin in a fibrin glue/fibronectin hydrogel significantly decreased the release of BMP-2 and prevented, effectively, the initial burst release. Additionally, the heparin cannot only influence the release kinetic, it also inhibits the in vivo-degradation of the cytokine by proteases [40,41]. In addition, it has been noted in the literature that tricalcium phosphate/hydroxyapatite bone substitutes coated with collagen type I and/or heparin show a higher loading capacity for BMP-2 and a different release pattern when the coating contains heparin. Thus, as might be expected, after 14 days, there is still BMP-2 bound in the layer system. For the samples made with collagen alone, almost all BMP-2 was released [42]. This can be explained by the enormous binding capacity of heparin which is also described for other cytokines and growth factors [43]. As demonstrated in this study, all the investigated PEMs contain heparin as a polyanion in each of the 10 double layers. So, this can be an indication of the observed low release of the BMP-2, insofar as the heparin binds the cytokine extremely stable and slows down the release kinetic. In this context, Laub et al. (2007) show the limited binding capacity of BMP-2 and other members of the TGF-β family to different collagen types [44]. Therefore, the high binding capacity of our multilayer systems is probably not mediated by the collagen, but rather through the heparin. An explanation, as to why the PEM3 system could be loaded with much more BMP-2 than the other two systems but releases less BMP-2, is the layer buildup. PEM3 is significantly thicker than PEM1 and PEM2 and shows an exponential growth because of the decreased intrinsic charge compensation and the open network structure, respectively, of the increased chain mobility within the PEM3 system. Beside the simple quantitative effect, it can be assumed that due to the weak cationic character of collagen, much more sulfate groups of heparin are available for the interaction with BMP-2 [45]. Hence, heparin is available much more freely within the PEM3 system, which then can act as a drug reservoir for BMP-2. This, consequently, increases the total loading capacity of the polyelectrolyte multilayer.

In this study, we observed the release under physiological conditions. Correspondingly, a conventional DMEM medium with a pH of 7.4 was used for all of the release experiments. Other studies have shown that the pH of the release medium influences the release of cytokines. Salvi et al. (2016) adsorbed around 2 µg/cm^2^ BMP-2 directly on anodized titanium covered with (PMAA/PLH)_5_. The release behavior was investigated in dependence of the pH value. The PEMs prepared at the lowest pH (pH = 4) show the highest BMP-2 release over 25 days. If the pH becomes more alkalic, the release decreases, reaching a minimum of around pH 6 and 7. This may reflect the fact that the change in the release kinetic results from changes in the internal structure of the PEM and the pH during preparation [46].

In any event, the investigated PEM systems here obviously serve much better as a drug reservoir than as a release system. This has, of course, some important consequences. For example, the incorporated drug is strongly bound in the matrix and is not delivered through diffusion caused by the concentration gradients in the media or the body fluids. In vivo cells adhere at the matrix and begin to modulate the cellular microenvironment, respectively, built up by their own ECM. This inevitably leads to a situation where the access to the cytokines is coupled to the rate of matrix degradation until the PEMs are fully degraded. In other words, in the case for bone regeneration, the BMP-2 is probably available for the entire bone healing process. Another fact is that BMP-2 delivery being higher than the clinically relevant doses might be associated with numerous complications, for instance tissue inflammation and abnormal or ectopic ossification. These complications result from the high doses which are needed to overcome short half-life and rapid clearance of the cytokine in vivo [30]. Thus, it can be speculated that a tailor-made PEM which serves primarily as a kind of BMP-2 drug reservoir is more advantageous than any burst release system. Hettiaratchi et al. (2020) investigated the positive effect of a drug reservoir for BMP-2 based on heparin microparticles [30].

### 3.2. Cell Response

To prove the cytocompatibility and suitability of the PEMs as a coating system for the better integration of implants in bone tissue, the PEMs were seeded with MC3T3-E1 cells, a mouse osteoblasts progenitor cell line, and the cell response was investigated thoroughly. The cell viability was measured by an XTT assay over a time period of 14 days.

**Figure 4:**
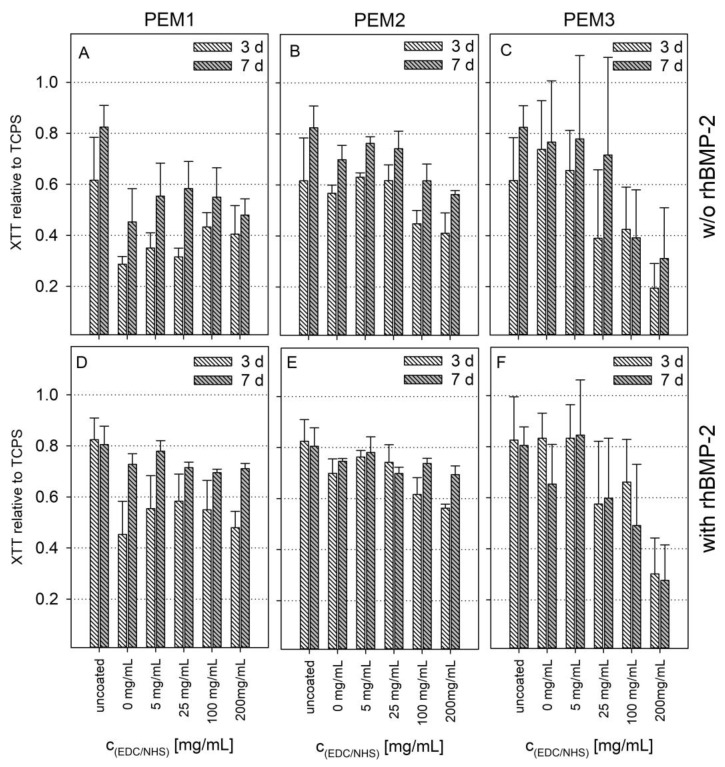
Absorbance in XTT assay relative to TCPS after 3 and 7 days on different cross-linked PEMs without BMP-2 (**A**–**C**) or loaded with BMP-2 (**D**–**F**). Error bars represent standard deviations of three independent experiments with three technical replicates each. Results of a one-way ANOVA test can be found in the Appendix A in Figure A3 and Figure A4.

Figure 4 show the measured absorption in the XTT assay for the time points 3 and 7 days without BMP-2 and with BMP-2 stimulation, relative to an uncoated tissue culture polystyrene reference (TCPS).

As shown in Figure 4A, the coating of PLL–HEP initially inhibits the cell viability at early time points. This effect can be explained by the reduced initial adhesion and proliferation of the cells on the relatively soft multilayers, as we have shown recently [36].

Cross-linking here results in a stiffening of the coating and thus an increased proliferation at EDC/NHS concentrations >100 mg/mL. The incorporation of a collagen top layer immediately leads to an increase in the viability of approximately 50% (Figure 4B). Strikingly, the cross-linking of the PEM2 coatings has negative effects above a certain concentration of EDC/NHS, which is particularly evident in the collagen-rich PEM3 system (Figure 4C).

On this background, it must be considered that cell binding to collagen is mediated by integrin binding sites. In the case of collagen, it is mainly the binding motif GFOGER on the helical collagen fiber which comes into play [23]. The EDC/NHS cross-linking chemistry obviously affects this binding site, because it forms amide bonds between primary amines and adjacent carboxylic groups. Thus, carboxylic groups which were modulated by the EDC/NHS cross-linking chemistry are no longer available as a bioactive group within the integrin binding motif. Indeed, this can result in a reduction in cell proliferation on EDC/NHS cross-linked collagen films [22]. This explanation is supported by the fact that cross-linking of the PEM2 coatings above an EDC/NHS concentration of 100 mg/mL causes a reduction in the viability, approximating the values of the corresponding PEM1 system, which has a similar coating architecture but does not contain the top collagen layer (Figure 4A,B).

However, for the non-cross-linked PEMs or the PEMs having a low degree of cross-linking, a positive effect on the cell viability was observed by introducing collagen type I.

BMP-2 loading generally leads to a slight but not significant increase in cell viability and partially compensates for the negative effects of cross-linking. Nevertheless, the influence of BMP-2 on the proliferation is weak, which is not surprising since BMP-2 is not a proliferation but a differentiation promoter.

### 3.3. Differentiation of MC3T3 into Bone Cells

#### 3.3.1. Early Differentiation

**Figure 5:**
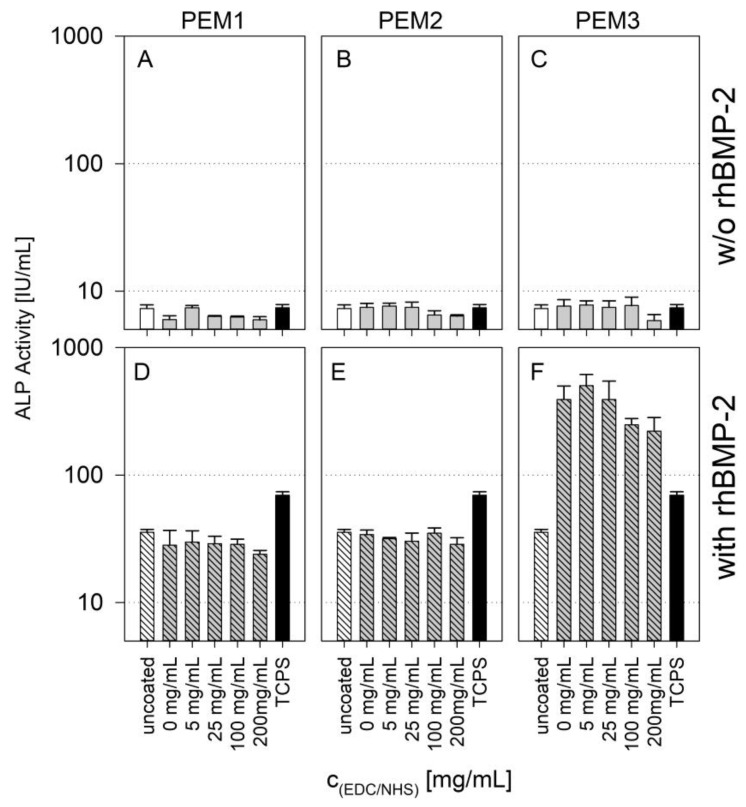
Results of ALP activity measurements after 7 days of cultivation in logarithmic scale; (**A**–**C**) without BMP-2; (**D**–**F**) with BMP-2. Results of a one-way ANOVA test can be found in the Appendix A in Figure A5.

The cells were cultured on different cross-linked PEMs loaded or non-loaded with BMP-2. After 7 days, the ALP activity was measured (Figure 5).

The presence of BMP-2 leads to an increase in ALP activity of about one order of magnitude for PEM1 and PEM2 as well as for uncoated glass and TCPS.

This is a relevant finding since the uncoated glass surface and the TCPS surface also shows this increase. The ALP activity was measured after 7 days, at a time when no release of BMP-2 was measurable on either the PEM coatings or the references.

However, the non-cross-linked coatings of the PEM1 and PEM2 systems show higher amounts of immobilized BMP-2 compared to the uncoated reference (Figure 3D and Figure 5). This means that the additional amount of cytokine immobilized in the PEM1 and PEM2 coating is obviously not bioavailable.

The PEM3 system shows a 10-fold increase in ALP activity compared to the PEM1 and PEM2 systems. There is a slight decrease in activity at high EDC/NHS concentrations, but this can be attributed to the already discussed reduced availability of binding motifs in the cross-linked collagen. Nevertheless, this increase in one magnitude of order can only be explained by the bioactive availability of the cytokine immobilized within the multilayer. The coating of heparin and collagen is apparently able to incorporate rhBMP-2 in its bioactive form. This observation was not made for PEM coatings containing less or no type I collagen.

#### 3.3.2. Late Differentiation

As osteogenesis progresses, the ECM acts, to an increasing extent, as a template for mineralization processes and more and more calcium is deposited to build up a calcified bone structure. Calcium deposition is a late marker of osteogenic differentiation. This process can be monitored with an Alizarin Red S Assay. The red dye Alizarin Red S binds to the calcium depositions in the matrix and can be removed with acetic acid. The more calcium that is incorporated in the matrix, the more dye that is released. Usually, this can be quantified by simple absorption measurements.

**Figure 6:**
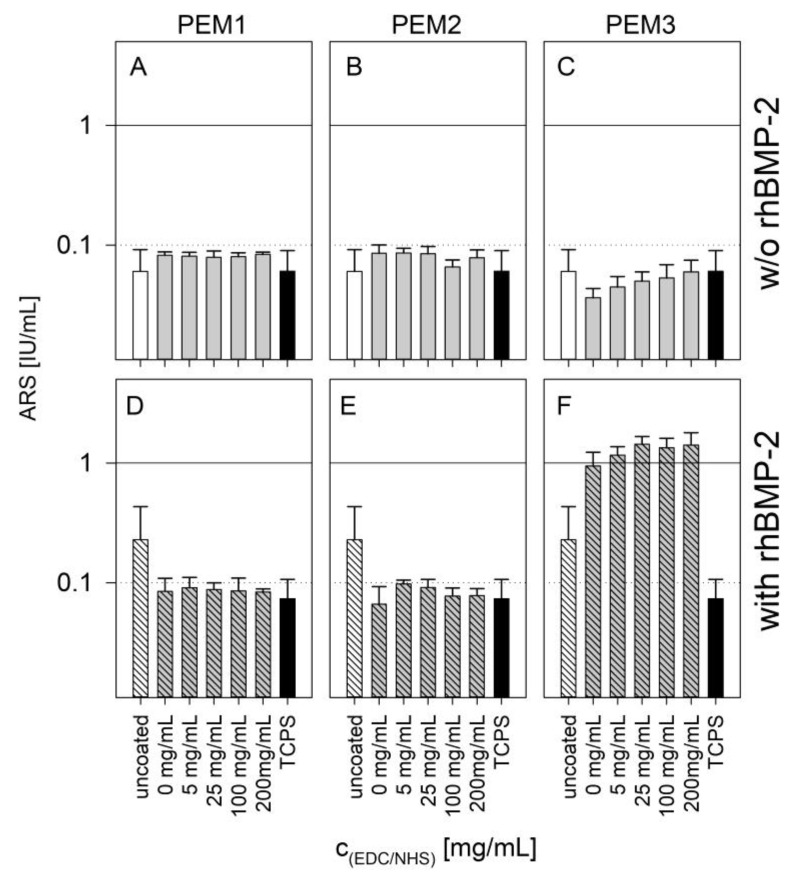
Absorbance of ARS assay on different cross-linked PEMs unloaded (**A**–**C**) or loaded with BMP-2 (**D**–**F**) after 21 days of cultivation in logarithmic scale. Error bars represent standard deviations of three independent experiments with three technical replicates each. Results of a one-way ANOVA test can be found in the Appendix A in Figure A6.

The results of the calcification by the Alizarin Red assay support the conclusions made on the basis of the ALP activity results (Figure 6). Both PEM1 and PEM2 coatings show no enhanced calcification after 21 days due to BMP-2 loading, whereas the PEM3 coatings show an enhancement of one order of magnitude.

Keeping in mind that the used MC3T3-E1 cell line is a specifically sensitive cell line to investigate surface-related effects on calcification, it can be stated that the late cell differentiation is strictly dependent on the bioavailability of immobilized BMP-2. This finding is in a good accordance with other studies which investigated the differentiation behavior of MC3T3-E1 cells on GAG/BMP-2-containing coatings [32,40].

Another effect shown by the results is the trend that PEM3 shows as a function of cross-linking. In both cases, with or without BMP-2 loading, an obvious correlation between the cross-linking and the ARS assay is evident. This effect might have mechanical reasons, as the cross-linked coatings show a drastic increased layer stiffness [36]. Nevertheless, it suggests that the immobilized rhBMP-2 cytokine plays a major key role concerning the observed differentiation behavior. These findings are supported by some other publications which focused on related topics [4,47,48].

## 4. Conclusions

The aim of this study was to investigate the performance of three LbL films with different multilayer compositions and architectures, regarding their suitability as drug reservoirs, respectively, and drug release systems. The investigations essentially focused on the effect of rhBMP-2 in bone contact. For this purpose, PEM systems were constructed on the basis of poly-L-lysine, a relatively strong poly-cation and heparin, a GAG of the ECM as well as collagen type I, a glycoprotein and one of the main components of the osteoid ECM. By gradually cross-linking the layers, we were also able to vary the stiffness of the multilayer films and, consequently, the release kinetics of rhBMP-2.

We found that a minor part of the incorporated rhBMP-2 was released from the coatings. Furthermore, the release occurred mainly within the first 48 h. We were able to confirm the hypothesis, that the release can be adjusted in a defined way by means of a cross-linking of the multilayers. In cell biological experiments, we referred to the differentiation of the osteoprogenitor cell line MC3T3-E1, however, we were able to demonstrate that neither the amount nor the kinetics of the release plays the primary role in osteogenic differentiation. Rather, BMP-2 immobilized in collagen-rich films was found to be highly bioactive. An impressive increase in ALP activity by two orders of magnitude after 7 d and an increase in calcification by one order of magnitude after 21 d were observed.

It must be concluded that polyelectrolyte multilayers made on the basis of heparin and collagen type I represent an excellent artificial ECM mimicry and a promising approach for the development of therapeutic options in bone regeneration and implantology.

## Figures and Tables

**Table 1:** Summarized results from physicochemical investigations as published and discussed in detail in [36].

	EDC	Roughness (AFM)				Thickness (Ellipsometry)		Amid-I (FTIR)		Young’s Modulus (AFM)		Wettability (CA H_2_O)	
	mg/mL	Sa [nm]	SD	Sdr [%]	SD	d [nm]	SD	Abs.	SD	E [kPa]	SD	CA [°]	SD
PEM1	0	0.68	0.10	0.01	0.00	70.07	3.75	0.24	0.03	-	-	28.07	2.20
	5	1.41	0.37	0.07	0.04	51.00	9.24	0.35	0.06	-	-	19.74	3.72
	25	1.38	0.10	0.04	0.01	42.37	16.21	0.37	0.06	-	-	14.60	4.50
	100	2.03	0.10	0.09	0.01	59.07	5.25	0.59	0.19	-	-	7.69	5.37
	200	3.61	1.21	0.32	0.21	61.33	11.56	0.78	0.38	-	-	46.96	0.99
PEM2	0	4.60	3.64	0.33	0.29	116.25	1.06	0.38	0.07	30.29	4.944	20.48	7.40
	5	1.90	0.21	0.10	0.02	115.95	0.92	0.52	0.07	279.8	66.7	10.27	7.96
	25	1.21	0.20	0.03	0.01	140.15	50.70	0.48	0.03	302	145.4	11.44	5.52
	100	7.14	1.91	0.38	0.19	117.30	14.42	0.59	0.06	248.2	131.2	12.81	3.62
	200	5.68	0.45	0.41	0.03	97.20	19.87	0.75	0.10	640.8	138.3	20.85	13.97
PEM3	0	7.32	2.88	0.26	0.07	227.00	111.72	0.88	0.19	19.6	5.302	20.48	8.40
	5	5.71	0.78	0.23	0.04	249.03	84.68	1.71	0.34	116.7	104.7	14.62	3.02
	25	5.63	0.59	0.26	0.03	327.50	44.55	1.45	0.39	245.1	103.6	15.14	6.34
	100	5.11	0.74	0.29	0.03	292.90	4.20	2.12	0.55	475.3	189.1	45.96	1.32
	200	2.09	0.39	0.16	0.04	233.90	73.54	3.36	1.12	600.7	88.95	50.62	3.23

**Figure A1:**
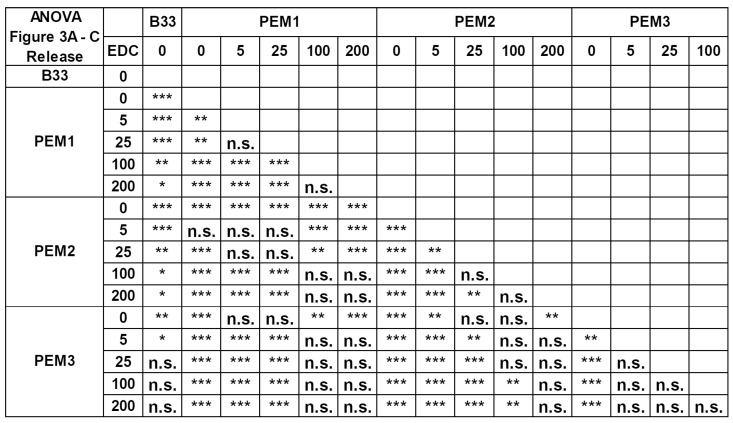
Results of one-way ANOVA for data related to Figure 3A–C; n.s.: not significant; *: *p* < 0.1; **: *p* < 0.05; ***: *p* < 0.001.

**Figure A2:**
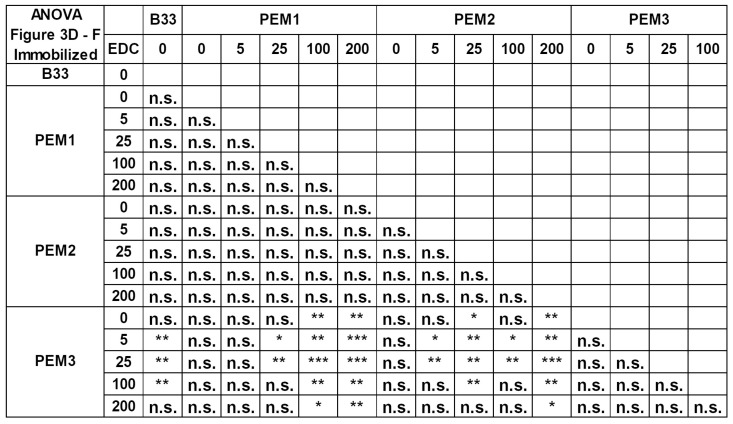
Results of one-way ANOVA for data related to Figure 3D–F; n.s.: not significant; *: *p* < 0.1; **: *p* < 0.05; ***: *p* < 0.001.

**Figure A3:**
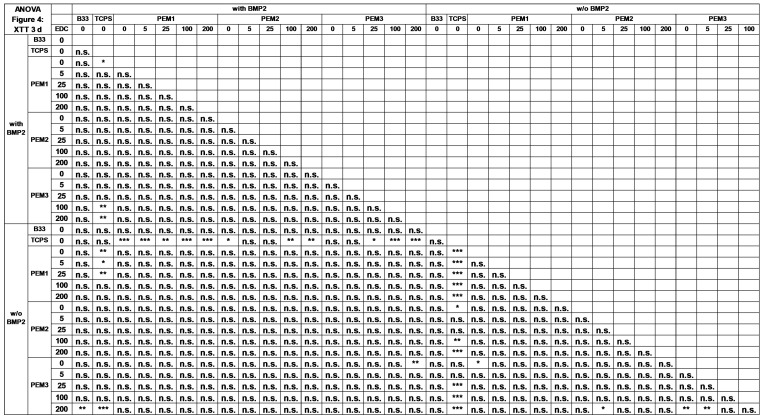
Results of one-way ANOVA for data related to Figure 4 (3 d); n.s.: not significant; *: *p* < 0.1; **: *p* < 0.05; ***: *p* < 0.001.

**Figure A4:**
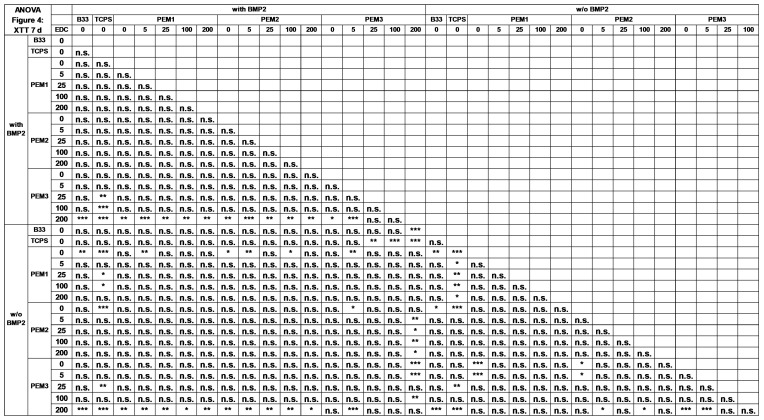
Results of one-way ANOVA for data related to Figure 4 (7 d); n.s.: not significant; *: *p* < 0.1; **: *p* < 0.05; ***: *p* < 0.001.

**Figure A5:**
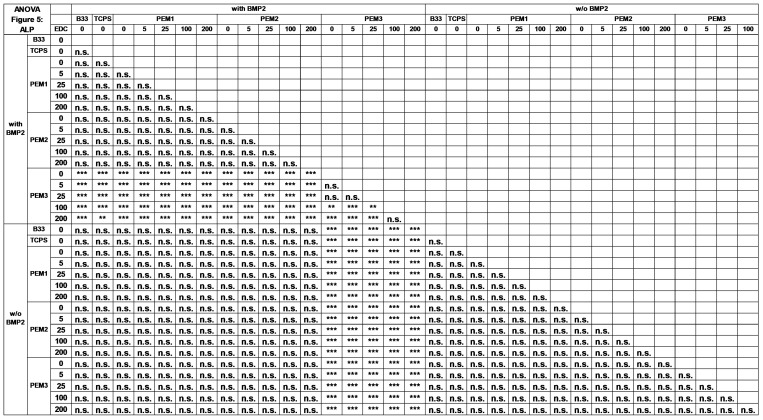
Results of one-way ANOVA for data related to Figure 5; n.s.: not significant; *: *p* < 0.1; **: *p* < 0.05; ***: *p* < 0.001.

**Figure A6:**
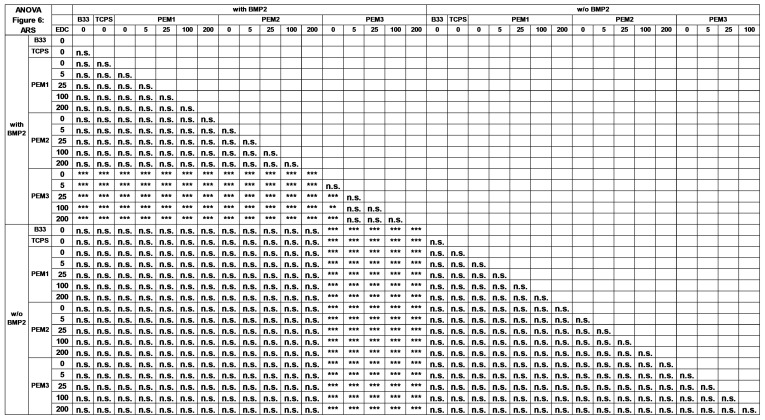
Results of one-way ANOVA for data related to Figure 6; n.s.: not significant; *: *p* < 0.1; **: *p* < 0.05; ***: *p* < 0.001.

## Data Availability

The data presented in this study are available on request from the corresponding author.
